# A float-controlled self-contained laser gauge for monitoring river levels in tropical environments

**DOI:** 10.1016/j.ohx.2025.e00682

**Published:** 2025-07-24

**Authors:** Alain Pierret, Norbert Silvera, Keo Oudone Latsachack, Khampasith Chanthavong, Phabvilay Sounyafong, Olivier Ribolzi

**Affiliations:** a GET UMR234, Université de Toulouse, CNRS, IRD, UPS, CNES, 31400 Toulouse, France; b IRD, IEES-Paris UMR 242, c/o Department of Agricultural Land Management (DALaM), Ban Nongviengkham, Xaythany District, Vientiane 01000 Lao PDR, France; c IRD, SMART Ban Lak Sip, Road 13, Luang Prabang, Lao PDR, France; d Mounoy Dev Xokham Village, Xaysetha District, Vientiane Capital, Lao PDR, France; e IRD, Department of Agricultural Land Management (DALaM), Ban Nongviengkham, Xaythany District, Vientiane, 01000 Lao PDR, France

**Keywords:** Hydrometry, Micro-controller, Data logging, Time-of-Flight, Ad hoc technology, Tropical environments

## Abstract

In this paper we present the design, construction and performance of a self-contained float-controlled water level gauge for monitoring water levels in streams and small rivers. This device is inexpensive (cost of about EUR 220), easy to build (no electronics skills or specialized tools required; assembled in a few hours) and straightforward to use. The gauge remains autonomously operational for several weeks in remote locations without the need for an external power supply or solar panel and in the harsh tropical climatic conditions. Data can be downloaded wirelessly in the field using an Android smartphone or tablet. This gauge is capable of a measurement precision of ±1 mm at temperatures ranging from 20 to 30 °C and accurate to within 2 mm of manual readings in a controlled laboratory environment. In the field, the mean absolute error (MAE) of measurements taken with the water level gauge compared to that obtained with the OTT-SE200 − a commercial float-controlled angle encoder water level gauge − over a full tropical rainy season and for a measurement range of 0.5 m, was 2.6 mm (n = 8,017).

## Highlights

• Very low cost, mechanical assembly from readily available PVC pipes, customizable to specific user needs.
• No need for external power supply or solar panels.
• Low power consumption and autonomy >2 months relying solely on 4x D-size batteries.
• Simple to operate, direct data download on Android smartphone with *Serial Bluetooth Terminal* app.
• Easy to maintain and repair.

## Graphical abstract

**Figure:**
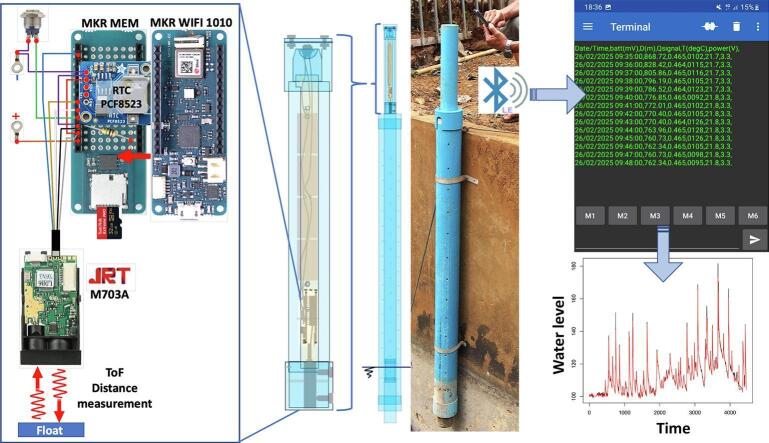

**Specifications table:** 

Hardware name	Float-controlled self-contained river level laser gauge
Subject area	Environmental, planetary and agricultural sciences
Hardware type	Field measurements and sensors
Closest commercial analog	No commercial analog is available
Open-source license	GPLv3
Cost of hardware	∼ EUR 220
Source file repository*	https://data.mendeley.com/datasets/dsffp9psmy/1

## 1 Hardware in context

Water level is a fundamental variable for estimating the discharge in a river section [1] and assessing the flow of dissolved and particulate matter [2], including contaminants such as pathogenic bacteria [3] and pesticide residues [4]. With the intensification of precipitation related to global climate change [5], monitoring river level is also crucial for controlling the propagation of flood waves along river systems and warning of the risk of flooding. Knowledge of river level is also essential for assessing the dynamics of the drying up of flows during periods of drought. In all these applications, accurate stream water level measurement in a large range is essential to limit calculation errors and anticipate the areas and periods at risk for ecosystems and populations downstream.

Although sensors capable of accurately measuring water level already exist on the market, they are generally expensive, fragile and ill-suited to the aggressive conditions encountered in rivers, particularly in the inter-tropical zone. Immersed sensors are exposed to suspended and bed load sediments and can show temporal drift. Sensors placed above the water surface may have moving mechanical parts that can wear out quickly, or electronic components that can be degraded by biological agents (e.g. biofouling).

In recent years, several authors have proposed several micro-computer/micro-controller based solutions for water level monitoring in the field, generally relying on ultrasonic rangefinders (e.g. [6], [7], [8]) or water level pressure transducers [9] powered by the mains grid, photovoltaic panels or high capacity batteries. Telemetric solutions based on IoT (Internet-of-Things) have also been proposed as a means to monitor water levels of rivers [10] water tanks [11] or sewer networks [12] in real-time.

In this paper we present details of the construction and qualification testing of a self-contained water level gauge based on visible laser Time-of-Flight (ToF) technology, designed as an integrated device for automatically measuring and recording water level data, that can be deployed to monitor changes in water level in streams and small rivers in tropical environments. The principles that guided us in designing the device were that the electronics and mechanics should be as simple as possible, using only the technology strictly necessary for the water gauge's intended use, without sacrificing metrological quality - what we hereafter refer to as *ad hoc* technology.

**Fig. 1:**
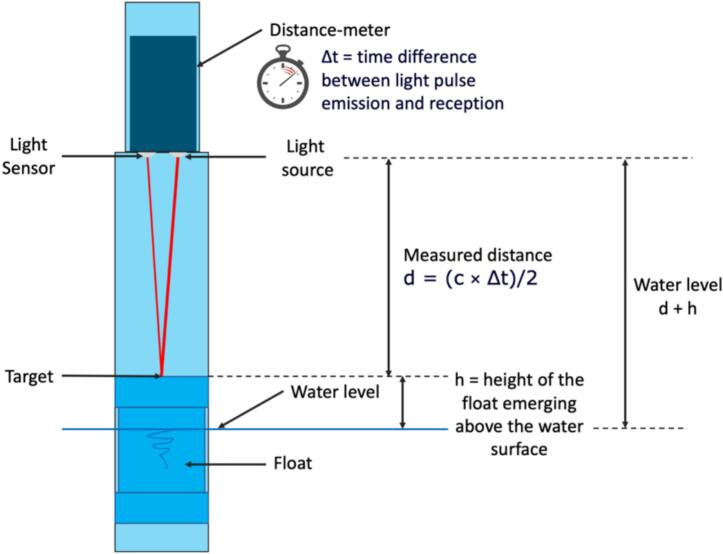
Schematic illustrating the principle of the water level measurement using a ToF laser distance meter.

We decided to use an optical ToF distance meter because, in principle, this technology offers a wider measuring range than that of ultrasonic rangefinders while their accuracy is higher, particularly at greater distances and their power consumption and Field-of-View (FoV) smaller [13]. The principle of optical Time-of-Flight (ToF) involves calculating distance based on the time it takes for light emitted by a source such as a laser and reflected off the surface of a target to return to a sensor. The delay Δt between the time the light is emitted and the time its reflection is received is used to determine the distance between the sensor and the target as d = (c × Δt)/2, where c is the speed of light. Next, h, the height of the float emerging above the water surface, is added to d to obtain the distance between the water surface and the distance meter (Fig. 1). Compared with ultrasonic-based measurements, optical ToF requires the use of a float as a reflective surface to materialize the water surface but the small target area corresponding to the narrow FoV is well-suited to such a configuration. A key specification of our water level gauge is that it can remain autonomously operational for several weeks in remote locations, in the harsh climatic conditions of the humid tropics and without the need for an external power source or solar panels.

## 2 Hardware description

### 2.1 General design

**Fig. 2:**
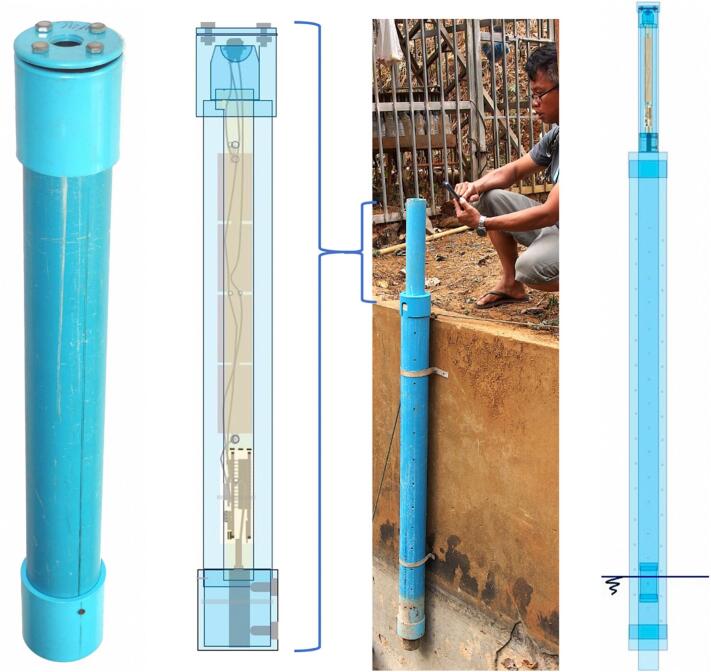
Overall view of the float-controlled self-contained water level gauge for the monitoring of stream levels. Far left: view of the complete gauge main housing with access to reset switch at the top. Left: transparent view of the gauge's mechanical and electronic components. Right: field setup for water level monitoring in a weir showing the strainer tube fixed to the wall of the weir and the long float guide tube at the top of which the gauge main housing is inserted. Far right: transparent view of the mechanical and electronic components of the field installation.

The self-contained water level gauge described and discussed in this paper has been designed to measure and record, over continuous periods of several weeks and at 1-minute time intervals, the level of a water surface within a range of 0.3 to at least 2 m in relation to a set reference, with a precision of the order of 1 mm, relying solely on an internal power supply (batteries) and involving as few mechanical parts as possible. All mechanical parts are manufactured from supplies that can be readily sourced locally in developing countries, namely PVC pipes, screws and clamps (no commercial, custom machined or 3D printed housing required). The overall arrangement of the device is shown in Fig. 2.

### 2.2 Main housing

The main housing of the water level gauge consists of a PVC tube hermetically sealed by two end caps: the lower end cap has two apertures through which the incident and reflected laser beams can pass (Fig. 2, Fig. 5, Fig. 13); the upper end cap has a central sealed aperture for access to the RESET push-button (Fig. 2, Fig. 5, Fig. 11). Three main components are found inside the main housing (i) the distance meter, centred using two adjustment bolts pushing against the walls of a longitudinally slotted PVC pipe, the whole being held together by a self-locking nylon cable tie; (ii) the battery pack / micro-controller cards holder, made of a two-third-round piece of PVC pipe, with 2 bolts being used as electrical contacts with the positive and negative poles of the battery pack (a flap is cut in the PVC pipe and plied between the batteries and the MKR boards to prevent accidental contact); and (iii) a PVC end cap that supports the RESET push-button and is fitted at the end of the battery pack / MKR cards holder.

### 2.3 Float

Water being transparent to laser light, water level reading is based on the reflection of the laser beam on a surface that materializes the water level. To this end, the water level gauge includes a float made out of a piece of PVC pipe hermetically sealed on both ends with PVC end caps and ballasted with a sufficient quantity of sand to ensure that it floats in water within ∼30 mm of its upper end. The height of the float emerging above the water surface has to be taken into account as a correction to the distance meter readings to obtain correct measurements of the water level.

### 2.4 Main assembly with double-walled supporting structure for water level monitoring in a weir

For the monitoring of water level in a weir, a large PVC tube 1.8 m long, serving as the main supporting structure and protecting the gauge + float assembly from sediment accumulation, was fixed along the wall of the weir. Regularly spaced holes were drilled in this tube, in the manner of a strainer tube, to allow unhindered water movement. Two end caps hollowed out to allow the passage of a smaller tube were added to this strainer tube. A 2.5 m long PVC pipe enclosing the main housing of the water level gauge at its upper end (held firmly in place using four 4 mm stop screws) was inserted in the strainer tube. It proved essential to drill several holes ø 5 mm near the upper end of this pipe to allow constant balance with atmospheric pressure as water level varies, in order to prevent buildup of pressure or depression that could affect the water measurements during rapid water level changes. Prior to inserting the float, it had to be checked that the laser pointer aimed at or close to the center of the top end cap of the float, particularly at the lower end of float guide tube (see section 5.6). Following the float insertion, a 4 mm stop screw was added at 10 mm from the lower end of the guide tube to prevent the float from falling off.

### 2.5 Distance meter

In this application we used the M703A laser distance meter from JRT (www.jrt-measure.com), a Time-of-Flight (ToF) visible laser module encapsulated in a sealed metal case protecting the optoelectronics from dust and water splashes. This distance meter has a measuring range of 40 m with an accuracy of 1 mm, an IP54 protection (as per manufacturer’s specifications) and a voltage requirement of 3.3 V.

### 2.6 Electronics

**Fig. 3:**
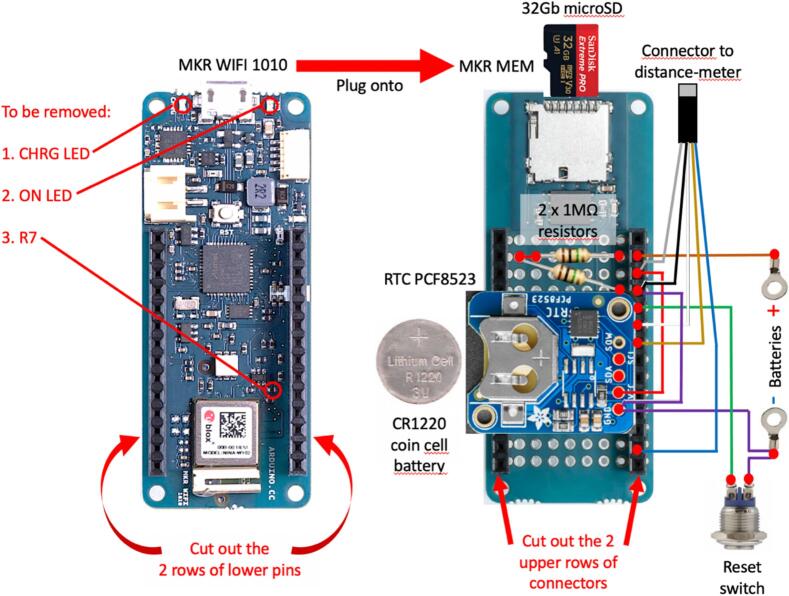
Overview of the electronics and wiring of the float-controlled self-contained water level gauge.

The electronics used to record data and communicate with the sensor are based on open-source platform components, including easy-to-use hardware requiring minimal soldering and simple programming language for customizing the device's functionality. To comply with such requirements, we used the Arduino MKR WIFI 1010 micro-controller (https://www.arduino.cc/) including a BlueTooth Low Energy (BLE) interface for wireless communication, stacked together with an MKR MEM SHIELD and connected to a RTC (Real Time Clock) PCF8523 from Adafruit (www.adafruit.com) powered by a CR1220 coin cell battery. The lower and upper connecting pins of the MKR WIFI 1010 and MKR MEM SHIELD, respectively, were cut to make the overall dimensions of the stack compatible with the space available in the main housing (see section 9.2). The MKR MEM SHIELD board hosts a 32 Gb micro-SD card and is connected to the RTC, 2x 1MΩ resistors, the distance meter connector, wires from the battery pack, and wires to a RESET push-button. The overall arrangement of the electronics is illustrated in Fig. 3.

### 2.7 Power supply and power efficiency optimization

The power supply consists of 4 × D-size 1.5 V batteries (non-rechargeable / 12,000 mAh from PANASONIC − battery capacity experimentally tested at ∼11,000 mAh by constant discharging with a current of 250 mA) so that the device is operable in any environment, including remote locations without the need for extra components such as solar panel, voltage regulator and rechargeable batteries that need maintenance (regular cleaning of solar panel) and could potentially attract the attention of malicious individuals. An important feature of the water level gauge being that it is intended to be deployed in remote areas, a special effort has been made to optimize power consumption to ensure several weeks’ autonomy with the four onboard D-size batteries. The proposed optimization relies both on hardware and software adjustments.

#### 2.7.1 Hardware mods

• Some non-essential components of the MKRZERO WIFI 1010 card need to be removed, namely the R7 resistor, ON LED and CHRG LED.
• The laser distance meter must be run with the standby option enabled (PWREN input enabled).
• BLE is only powered on after pressing the RESET push-button. At the office, use the device as much as possible connected to an external power source via the USB connector. Power the device on batteries only before installing on site.

#### 2.7.2 Software optimization

Specific instructions and library were used to reduce power consumption, namely:

• A soft restart following data acquisition to disable the BLE interface (instruction NVIC_SystemReset()).
• The use of an optimized library for SD card management (SdFat.h).
• Input mode configuration of unused micro-controller pins (pinMode(x,INPUT_PULLUP);).
• The use of several small files to reduce the time for downloading.

### 2.8 Program and wireless communication

The program (Table 1 *&* Fig. 20) is written with the open-source software IDE 1.8.16 from Arduino and supports data acquisition at a 1-min time step, recording to SD memory, RTC management and MKR sleep mode of WIFI 1010 between measurements (see *Flowchart –* Fig. 20). The iterative data acquisition sequence includes 1. Powering up the distance meter for distance and temperature measurements, 2. Saving the data on the SD memory card and 3. Putting the micro-controller to standby mode until the next iteration. Configuration is accessible via BLE communication (see Section 3) using a smartphone running the ‘Serial Bluetooth Terminal’ Version 1.48 application for Android written by Kai Morich (available from Google Play Store or https://www.kai-morich.de/android/).

**Table 1:** List of the design and source files.

Design file name	File type	Open source license	Location of the file*
SCWL gauge electronic design April 2025	.pdf	*GPLv3*	https://data.mendeley.com/datasets/dsffp9psmy/1
SCWL gauge mechanical design April 2025	.pdf	*GPLv3*	https://data.mendeley.com/datasets/dsffp9psmy/1
LoggerMKR0lp8e	.ino	*GPLv3*	https://data.mendeley.com/datasets/dsffp9psmy/1
RTC_PCF8523	.cpp	*GPLv3*	https://data.mendeley.com/datasets/dsffp9psmy/1
RTClib	.h	*GPLv3*	https://data.mendeley.com/datasets/dsffp9psmy/1
HardwareBLESerial	.cpp	*GPLv3*	https://data.mendeley.com/datasets/dsffp9psmy/1
HardwareBLESerial	.h	*GPLv3*	https://data.mendeley.com/datasets/dsffp9psmy/1

In Table 1 are listed the design files that are associated with the project.

• SCWL gauge electronic design April 2025.pdf includes the gauge's electronic circuit diagram, as well as an electronic schematic and an alternative circuit diagram to implement an ultra-low-power version of the device.
• SCWL gauge mechanical design April 2025.pdf includes a detailed description of the components of the self-contained gauge assembly, as well as mechanical drawings with dimensions to guide the construction of the self-contained water level gauge.
• LoggerMKR0lp8e.ino is the source code to be uploaded to the MKR wifi 1010 card using the Arduino IDE software in order to operate the water level gauge.
• ‘RTC_PCF8523.cpp’, ‘RTClib.h’, ‘HardwareBLESerial.cpp’ and ‘HardwareBLESerial.h’ are libraries with modifications required to run the ‘LoggerMKR0lp8e’ program.

## 3 Bill of materials summary

The List of materials used to build the gauge with indicative prices and potential suppliers is given in Table 2. Additional materials/equipment required to assemble the water level gauge:

• Soldering iron
• Cable cutters
• Cutter and cutting board
• Silicone sealant
• PVC glue
• Sand
• Pencil, pen, marker
• Metal saw
• Drill, 2/4/5/6 mm and step cone drill bit
• Phillips screwdriver / M6 spanners
• USB to micro-USB cable

**Table 2:** List of materials with indicative prices as per April 2025 and potential suppliers. Prices are exclusive of taxes. *Reference numbers in brackets in the ‘Designator’ column correspond to parts referred to in Fig. 4, Fig. 5, Fig. 6, Fig. 7, Fig. 8, Fig. 9, Fig. 10, Fig. 11, Fig. 12, Fig. 13, Fig. 14, Fig. 15, Fig. 16, Fig. 17, Fig. 18, Fig. 19; ^1^price per linear meter; ^2^sold by 10 units; ^3^price for 50 g; ^4^sold as packs of 2 units; ^5^sold in 4 m length; ^6^sold as packs of 100 pieces.

Designator*	Component	Number	Unit Cost in €	Total cost in €	Source of materials	Material type
Laser distance meter (#8)	JRT M703A distance meter + cable + cover	1	62.00	62.00	https://www.jrt-measure.com	Electronic
Micro-controller card (#14)	MKR WIFI 1010	1	33.25	33.25	https://www.gotronic.fr	Electronic
Micro-controller shield (#14)	MKR MEM	1	21.92	21.92	https://www.gotronic.fr	Electronic
Memory card (#11)	32 Gb micro-SD card	1	14.92	14.92	https://www.gotronic.fr	Electronic
Real time clock (#14)	RTC PCF8523 Adafruit	1	6.92	6.92	https://www.gotronic.fr	Electronic
Reset push-button (#16)	(ON)-OFF push-button	1	3.83	3.83	https://www.gotronic.fr	Electronic
Electric wire (#15)	wire 0.2-mm^2^ section (3 colors)^1^	3	0.25	0.75	https://www.gotronic.fr	Electronic
Resistor (#14)	1MΩ 0.25 W resistor^2^	2	0.02	0.04	https://www.gotronic.fr	Electronic
solder	Tin/lead solder^3^	1	4.12	4.12	https://www.gotronic.fr	Electronic
coin cell battery (#13)	CR1220 coin cell battery	1	1.42	1.42	https://www.gotronic.fr	Battery
D-size battery (#15)	D-size batteries^4^	4	6.30	12.60	https://www.homepro.co.th	Battery
Tube (#9, 10, 19)	55 (2″)/OD:60 mm PVC pipe^5^	1	3.20	3.20	https://www.homepro.co.th	PVC plumbing conduit
Tube end cap (#1, 4, 7)	55 mm (2″) – length 71 mm/PVC end cap	3	0.80	2.40	https://www.homepro.co.th	PVC plumbing conduit
Tube end cap (#18, 20)	55 mm (2″) – length 28 mm/PVC end cap	2	0.80	1.60	https://www.homepro.co.th	PVC plumbing conduit
Tube (#15)	31 mm (1.1/4″) OD:34 mm PVC pipe^5^	1	6.70	6.70	https://www.homepro.co.th	PVC electrical conduit
Tube end cap (#17)	34 mm (1.1/4″) − length 46 mm PVC end cap	1	0.60	0.60	https://www.homepro.co.th	PVC plumbing conduit
Tube (#22)	65 (2.1/2″)/OD:76 mm PVC pipe^5^	1	5.30	5.30	https://www.homepro.co.th	PVC plumbing conduit
Tube end cap (#21)	65 mm (2.1/2″) PVC end cap	1	1.60	1.60	https://www.homepro.co.th	PVC plumbing conduit
Tube (#25)	125 (5″)/OD:140 mm PVC pipe^5^	1	26.50	26.50	https://www.homepro.co.th	PVC plumbing conduit
Tube end cap (#23, 24)	125 mm (5″) PVC end cap	2	4.50	9.00	https://www.homepro.co.th	PVC plumbing conduit
Tube accessory	125 mm (5″) mounting pipe clamps	2	1.5	3.00	https://www.homepro.co.th	PVC plumbing conduit
Fastener (#2, 12)	Self-locking cable tie (w x l: 2.5 x 100 mm)^6^	2	0.70	0.01	https://www.homepro.co.th	Fastener
Hex bolts and nuts (#6, 9)	M6 x 40 mm bolts and nuts	10	0.09	0.90	https://www.bricovis.fr	Fastener
Screws (#3, 22)	Self-drilling screws 2.9 x 13 mm	5	0.06	0.30	https://www.homepro.co.th	Fastener

## 4 Build instructions

### 4.1 Mechanical making

#### 4.1.1 Distance meter unit holder #9 (Fig. 4, Fig. 5, Fig. 8)

i. Cut a 70 mm length of int ø 55 mm/ext ø 60 mm PVC pipe.
ii. Cut off a 16 mm wide, 70 mm long slit on one side of this tube.
iii. Drill 2 x ø 6 mm holes opposite to the slit.
iv. Hollow out a 30x7 mm space, centered on the axis of the slit, at one end of the tube.
v. Cut two ø 6 mm bolts to a length of 14.5 mm, insert them into the holes drilled at step (iii) and tighten them with 2 nuts.

#### 4.1.2 Electronic module holder #15 (Fig. 4, Fig. 6, Fig. 9)

i. Cut a 430 mm length of int ø 31 mm/ext ø 34 mm PVC pipe.
ii. Cut off one-third of its circumference to a length of 413 mm.
iii. Drill 2 × ø 6 mm holes perpendicular to the tube at 128 mm from one of its ends (to insert the bolt to which the (+) battery terminal must be attached).
iv. Drill 1 × ø 6 mm hole at 374 mm from the same end of the tube as in (iii) on one side of the tube and at 386 mm on the other side (to insert the bolt to which the (−) battery terminal must be attached.
v. Drill 4 × ø 3 mm holes for cable routing (from RESET push-button and (−) terminal);
vi. Cut a 25 mm long × 20 mm wide flap in the lower part of the tube and fold it up at 90° to isolate the electronic module from the (+) terminal.

#### 4.1.3 RESET push-button cap #17 (int ø 34 mm, ext ø 42 mm, length 47 mm) (Fig. 4, Fig. 5, Fig. 11)

i. Drill 1 × ø 16 mm hole centered on the end of the cap;
ii. File 4 beveled sides 32 × 15 mm.

#### 4.1.4 Distance meter module cap #1 (int ø 60 mm, ext ø 70 mm, length 71 mm) (Fig. 4, Fig. 5, Fig. 13)

i. Drill 1 × ø 10 mm hole near the center of the cap and 1 x ø 6 mm hole next to it.

#### 4.1.5 Reset push-button insulating cover holder #7 (int ø 60 mm, ext ø 70 mm, length 71 mm) (Fig. 4, Fig. 5, Fig. 12)

i. Drill 1 × ø 20 mm hole centered on the end of the cap-
ii. Drill 4 × ø 6 mm holes, 90° apart, 25 mm from the center of the cap.

#### 4.1.6 Reset push-button insulating cover #4 (made from cap int ø 60 mm, ext ø 70 mm, length 71 mm) (Fig. 4, Fig. 5, Fig. 12)

i. Cut out the flat end of the cap to a thickness of ∼5 mm-
ii. Drill 1 x ø 20 mm hole centered on the end of the cap-
iii. Drill 4 x ø 6 mm holes, 90° apart, 25 mm from the center of the cap (and matching that of #7).

#### 4.1.7 Rubber membrane for reset push-button #5 (Fig. 4, Fig. 5, Fig. 12)

i. Cut a piece of rubber 66 mm in diameter from a used inner tube, for example;
ii. Cut out 4 holes ø 6 mm 90° apart, 25 mm from the center of the cap (matching that of #4 and #7).

#### 4.1.8 External enclosure #10 (Fig. 4, Fig. 5, Fig. 13)

i. Cut a length of 426 mm of int ø 55 mm/ext ø 60 mm PVC pipe.
ii. Drill 1x ø 3 mm hole at 10 mm from one end of the tube.

### 4.2 Electronic making (Fig. 3, Fig. 7)

i. Take out the 2 lower rows of connector pins from the MKR WIFI 1010 board with wire cutter. Using a wire cutter, remove the On LED, the CHRG LED and resistor R7.
ii. Take out the 2 upper rows of female connectors from the MKR MEM with wire cutter.
iii. On the MKR MEM board, solder one 1MΩ resistor between DAC0/A0 and VIN and one 1MΩ resistor between DAC0/A0 and GND (Fig. 7*a*).

**Fig. 4:**
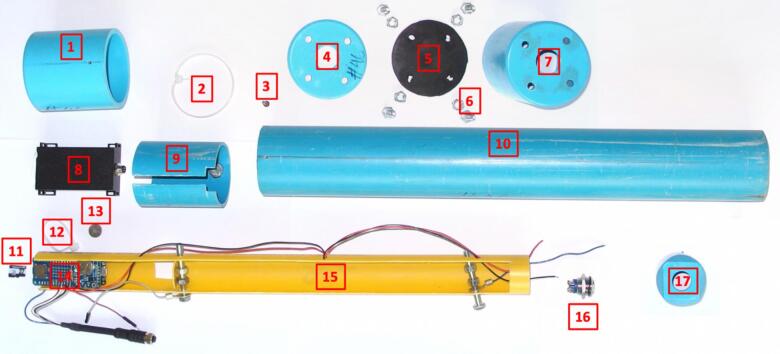
Parts of the self-contained gauge assembly #1 – Distance meter module cap; #2 − self-locking cable for distance meter unit holder; #3 − Locking screw; #4 − Reset push-button insulating cover; #5 − Rubber membrane for reset push button; #6 − Bolts and nuts set for reset switch insulating cover; #7 − Reset push-button insulating cover holder; #8 − Distance meter unit; #9 – Distance meter unit holder; #10 − External enclosure; #11–32 Gb micro-SD card; #12 − self-locking cable for electronics assembly; #13 − CR1220 coin cell battery; #14 − Electronics assembly; #15 − Electronic module holder; #16 − Reset push button; #17 − Reset push-button cap.

**Fig. 5:**
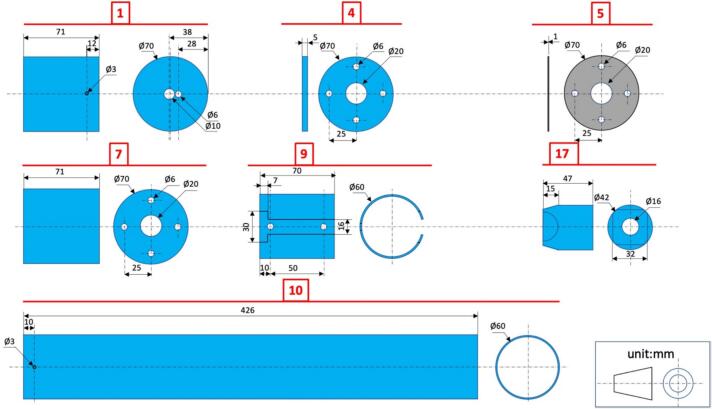
Drawings with dimensions of the PVC parts used for the construction of the self-contained water level gauge assembly.

**Fig. 6:**
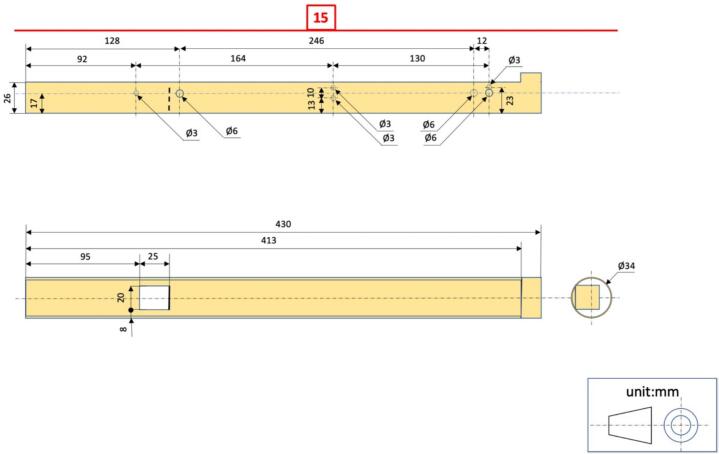
Drawings with dimensions of the PVC parts used for the construction of electronic module holder.

**Fig. 7:**
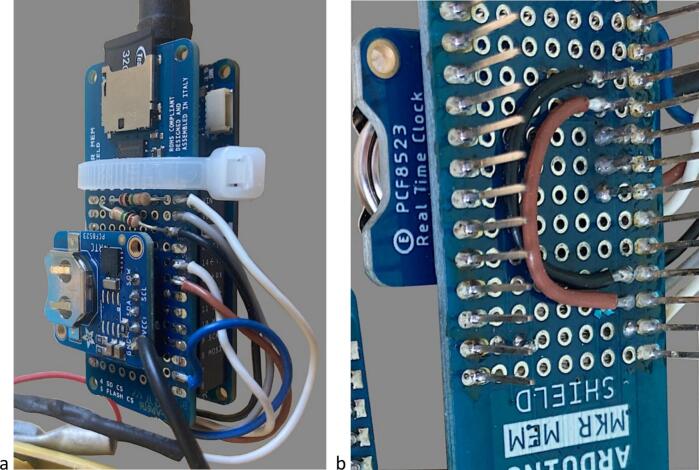
Electronics assembly. a. Overall view of the electronics assembly #14 showing the RTC module to the top left, the MKR MEM card with 32 Gb micro-SD card inserted in the micro-SD drive, 2x 1MΩ resistors and electrical wires soldered and the self-locking cable #12 securing it on top of the MKR WIFI 1010 card; b. Detailed view of soldering GND and VCC pins of RTC module under the MKR MEM card.
iv. Solder 4 connection pins to the RTC.
v. Solder the SDA and SCL pins of the RTC to the correspond pins of the MKR MEM and bridge GND and VCC pins with two short wires under the MKR MEM card (Fig. 7*b*).
vi. Strip and tin the 5 wires of the distance meter cable.
vii. Solder wires of the distance meter to the MKR MEM board pins: black to GND, grey to VCC, brown to RX, white to TX, blue to 7.
viii. Solder the (+) cable from the VIN pin of the MKR MEM to the (+) lug terminal.
ix. Solder a cable to the GND of the RTC at one end and to the (−) lug terminal at the other end.
x. Solder the RESET cable to the RESET signal of the MKR MEM.
xi. Tighten the 2 electronic cards with a self-locking cable tie #12.
xii. Pass the (−) and RESET cables through the 4 small holes of the #15 PVC tube (Fig. 6, Fig. 9c,d).
xiii. Solder a short piece of cable to the (−) lug terminal.
xiv. Apply a bead ∼20 mm × 30 mm of silicon sealant around the two windows of the laser distance meter (Fig. 8*f*).

**Fig. 8:**
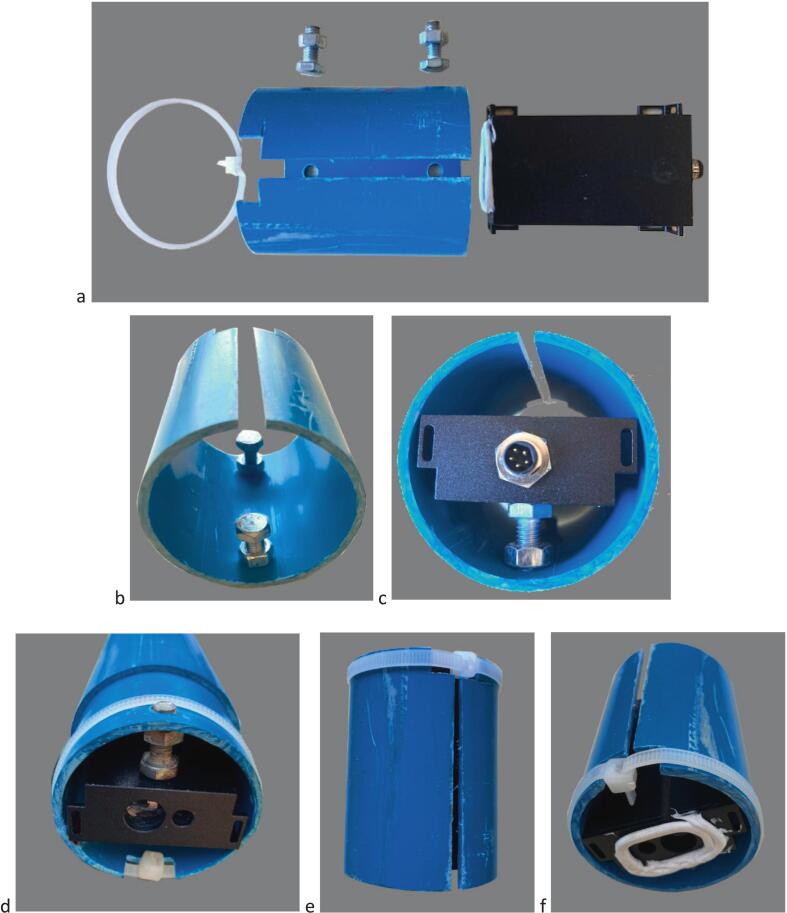
Installation of the distance meter unit in its holder. a. Parts of the distance meter unit holder, including self-locking cable #12 (left), distance meter unit holder #9 with ø 6 mm bolts and nuts (center), #8, distance meter unit (right); b. Internal view of the distance meter unit holder #9 showing the position of the distance meter fixing bolts; c. Rear view of the distance meter unit holder#9 with the distance meter unit #8 installed; d. Front view of the distance meter unit holder #9 with the distance meter unit #8 installed and the self-locking cable #2 sufficiently tightened so that the holder #9 can be inserted at one end of the external enclosure #10 of the gauge assembly; e. External view of the distance meter unit holder #9 showing the position of the self-locking cable #2 on the side of the cut-out slit; f. Front view of the distance meter unit holder #9 with the distance meter #8 unit installed and the bead of silicon sealant applied around its two optical windows.

### 4.3 Assembling the gauge unit 1/2

#### 4.3.1 Laser distance meter holder #9 (Fig. 4, Fig. 5, Fig. 8)

i. loosely fasten the self-locking cable #2 tie around the distance meter unit holder #9.
ii. Insert the 2 short ø 6 mm bolts from the outside of the holder #9 and screw the 2 nuts onto the inside of the holder (Fig. 8*b*).
iii. Insert the laser distance meter in the holder #9.
iv. Adjust the 2 bolts and the #2 self-locking cable so that the distance meter is firmly attached to the holder (Fig. 8*c,d*).

#### 4.3.2 Electronic module holder (Fig. 9)

**Fig. 9:**
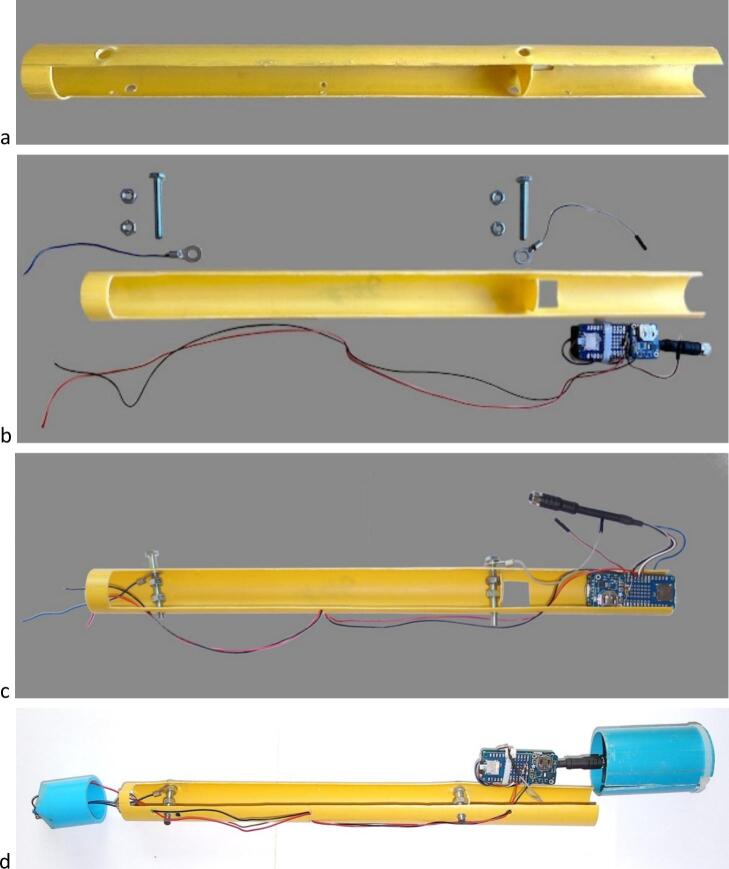
Electronic module holder construction and wiring. a. overall view of the electronic module holder #15 showing cut-outs and positioning of holes for bolts and electric wires; b. components to be assembled with the electronic module holder #15, including the electronics assembly #14, contact bolts and nuts, lug terminals and electric wires; c. view of the electronic module holder #15 after the electrical wires have been laid; d. view of the electronic module holder after the electrical wires have been laid and with the reset push button #16 connected and installed in reset push-button cap #17 and with electronics assembly #14 connected to the distance meter #8 installed in the distance meter unit holder #9.

**Fig. 10:**
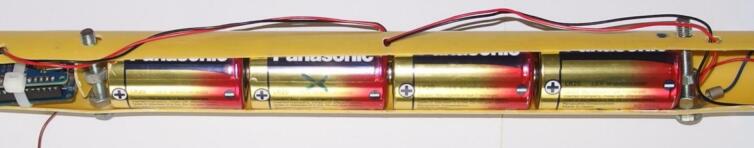
View showing the installation of the batteries in the electronic module holder #15 and the detail of electrical contacting using bolts and nuts.
iii. Insert the second long screw, 2 nuts and lug terminal according to the procedure described under (i). Adjust the second nut to ensure electrical contact between the 4 batteries and the two terminals.
iv. Insert the short cable of the (−) terminal and the RESET push-button cable through the push-button tightening nut and the RESET push-button cap #17 (Fig. 11*d*);

i. Insert the (+) lug terminal on one of the two long screws; insert the screw in one of the holes close to the cut-out flap, screw on 2 nuts, insert the screw in the hole on the opposite side of the tube and tighten the bolt against the tube on the lug terminal side (the second nut is used to make electrical contact with the (+) of the battery) (Fig. 9*a,b,c*).
ii. Insert the 4 batteries with the + terminal in contact with the nut screwed to the screw installed in step (i) (Fig. 10).

**Fig. 11:**
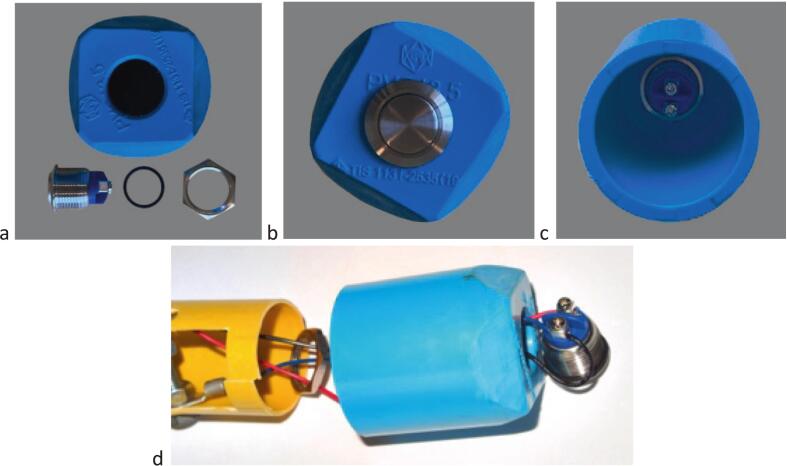
Assembling of the reset push-button. a. Components of the reset push-button including the push-button cap #17 (top), the push-button #16 (bottom left), its O-ring (bottom centre) and tightening nut (bottom right); b. External view of the reset push-button; c. Internal view of the reset push-button; d. Detail of reset push-button #16 wiring and insertion into its cap #17.
v. Solder the 2 cables to the switch with O-ring seal.
vi. Screw the reset push-button to cap #17.
vii. Gently plug the distance meter paying attention to the connector keying with 2 flat parts: push the connector then screw on the metal collar until it is tight (Fig. 13*a*).

#### 4.3.3 Reset push-button cap and cover (Fig. 12)

i. Sandwich membrane #5 between cap #7 and cover #4, then secure the assembly using set #6 of 4 screws + nuts.

### 4.4 First startup

i. Format the 32 Gb micro-SD card #11 in FAT format (Fig. 4);
ii. Insert the 32 Gb micro-SD card #11 in the MKR MEM #14 micro-SD slot (Fig. 4, Fig. 7);
iii. Insert the CR1220 coin cell battery #13 in the RTC battery holder (+ side facing upwards) (Fig. 4, Fig. 7);
iv. Connect the MKR WIFI 1010 to the computer with the USB / micro-USB cable;
v. Double click on the reset push-button to configure the MKR WIFI 1010 to receive a new program (the yellow LED is flashing slowly);
vi. Upload the program via the Arduino IDE:
• File > Open > LoggerMKR0lp8e.ino.
• Tools > Board > Arduino MKR WiFi 1010.
• Tools > Ports and choose the port to which the micro-controller is connected.
• Sketch > Upload or click on the right arrow on the window.
vii. Connect to an Android smartphone using the Bluetooth Low Energy interface (BLE − see paragraph 6.3):
• Check the power from D batteries using the p command.
• Check the RTC battery using the b command.
• Name the device using the n command.
• Write a header to the SD with h command (in file #00 by default).
• Set the date and time with t command.
• Check the date and time with d command.
• Check the distance meter with lD command.
• Start an RTC drift observation with a command.
• Let the device running up to 1 week, powering the MKR WiFi 1010 card either with batteries or via its micro-USB socket.
• After this 1-week observation period, put back the batteries or replace with new set of batteries and calibrate the RTC with c command.
• Check the calibration of the device with o command.
• The device is ready to start with m command.

### 4.5 Assembling the gauge unit 2/2 (Fig. 13)

i. Insert the electronic module holder #15 in the external enclosure #10.
ii. Gently insert the distance meter unit holder #9 onto the end of enclosure #10 to a depth of ∼ 1 cm (Fig. 13*d*).

**Fig. 12:**
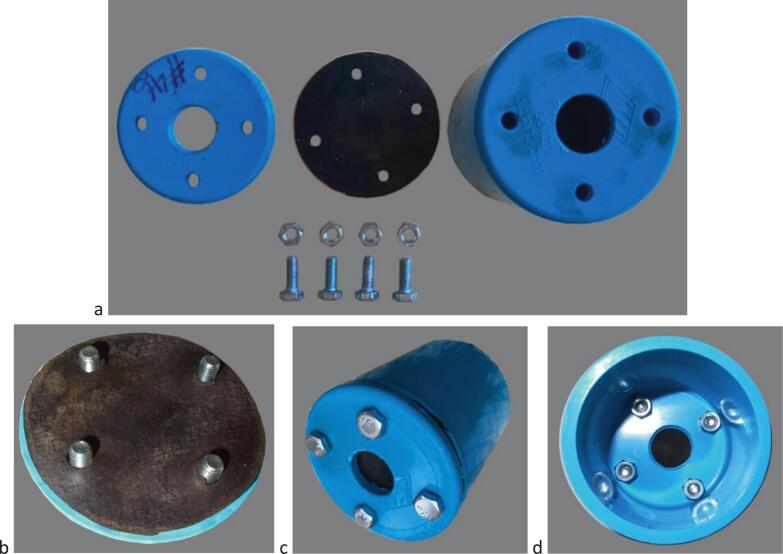
Assembling of the reset push button cap with insulating cover; a. Components of the reset push button cap and cover including the reset push-button insulating cover #4 (top left), the rubber membrane for reset push button #5 (top center), the reset push-button insulating cover holder #7 (top right) and the ø 6 mm bolts and nuts #6; b. Positioning of the rubber membrane and bolts on the insulating cover #4; c. External view of the push button cap after assembly showing the rubber membrane sandwiched between the insulating cover #4 and the insulating cover holder #7; d. Internal view of the push button cap after assembly showing the position of the tightening nuts.

**Fig. 13:**
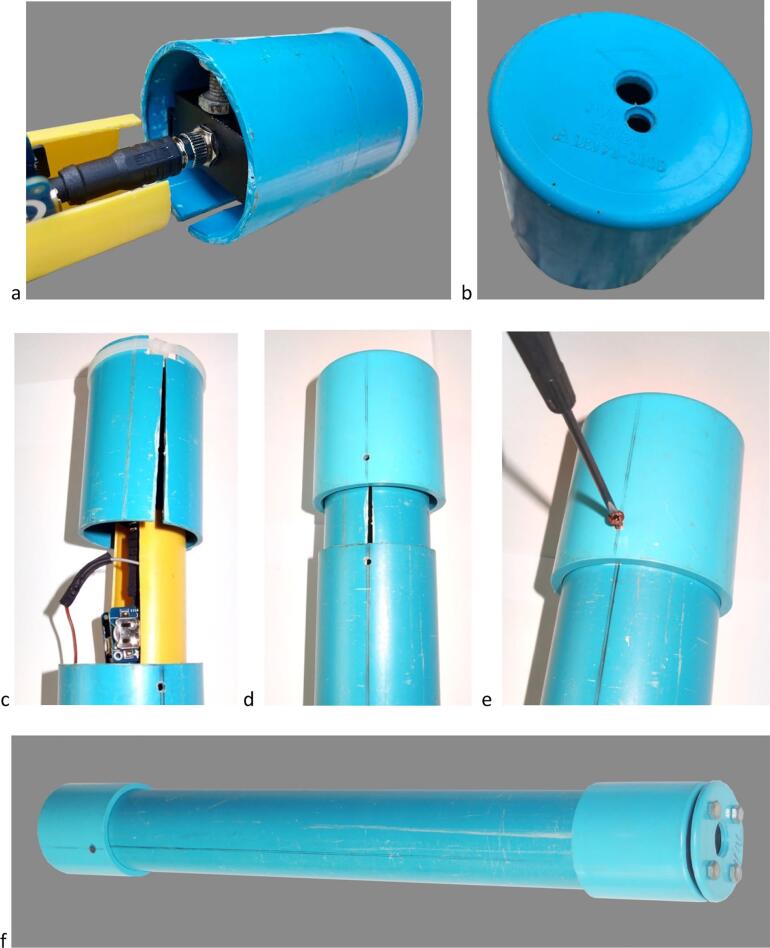
Final assembly of the gauge unit. a. Close-up showing the end of the electronic module holder #15 (left), the distance meter connector with the metal collar screwed tight (center) and the distance meter unit holder #9 (right); b. External view of the distance meter module cap #1 showing the holes to be aligned with the distance meter emitter and sensor windows when attaching #1 to the external enclosure #10; c. Insertion of the electronic module holder #15 into the external enclosure #10, ensuring slot in the distance meter unit holder #9 is aligned with the hole drilled on external enclosure #10 to receive the locking screw #3; d. Insertion of the distance meter module cap #1 onto the external enclosure #10; e. Final locking of the distance meter unit using the locking screw #3; f. Overall view of the complete gauge unit with distance meter module cap #1 to the left and reset push-button insulating cover to the right.

**Fig. 14:**
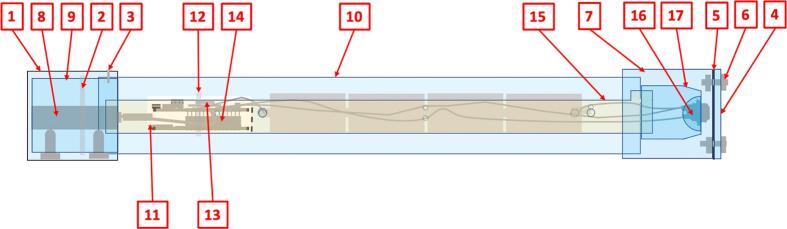
Transparent view of the fully assembled gauge, with indication of the reference numbers for mechanical and electronic components (see Fig. 4 for parts list).

**Fig. 15:**
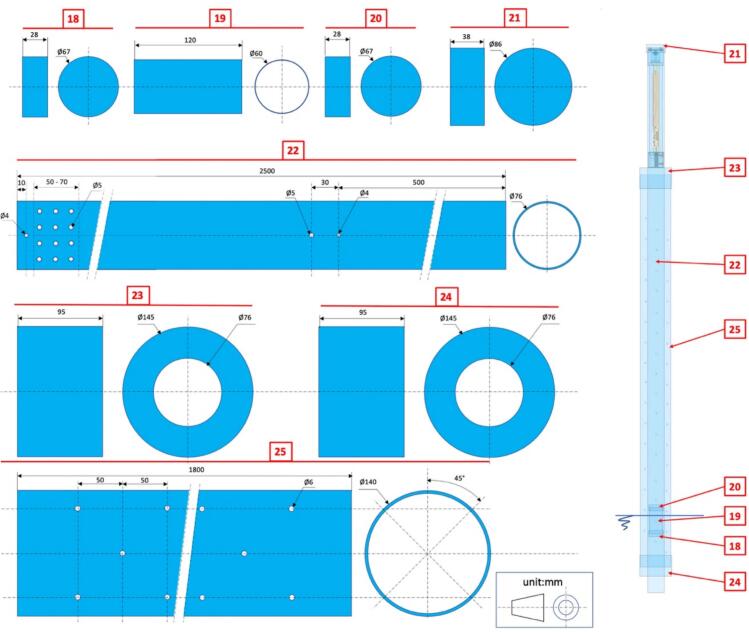
Left: Drawings with dimensions of the PVC parts used for the field setup for water level monitoring in a weir. #18 − Float lower end cap; #19 − Float body; #20 − Float upper end cap; #21 − Removable end cap for #22; #22 − Float guide tube; #23 − End cap for #25; #24 − End cap for #25; #25 − External strainer tube. Right: Transparent view of the fully assembled water monitoring device as installed in a weir, with indication of the reference numbers for mechanical components.
iii. Insert the distance meter module cap #1 and turn to adjust the 2 holes in front of the 2 windows of the distance meter (Fig. 13*d*); Fix the locking screw #3 to maintain the distance meter holder in the external tube (Fig. 13*e*).
iv. Insert the cap reset push-button side (#4+#5+#7 assembly) (Fig. 13*f*).

### 4.6 Mechanical making for water level monitoring in a weir (Fig. 15, Fig. 16, Fig. 17, Fig. 18)

**Fig. 16:**
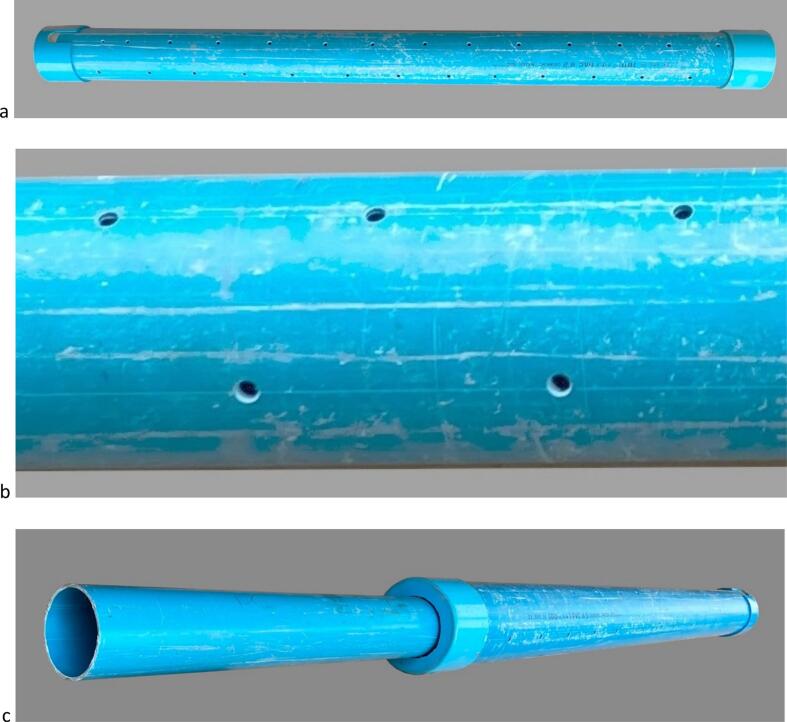
PVC parts for water level monitoring in a weir; a. Overall view of external strainer tube #25 with end caps #23 and 24 inserted; b. Detail of external strainer tube #25 showing holes regularly drilled to allow for free water circulation; c. Overall view from the top side of the float guide tube #22 inserted in the external strainer tube.
iii. Cut a ø 76 mm diameter circular opening in the centre of two ø 145 mm diameter PVC end caps (#23 and #24) and glue them to the ends of tube #25; the circular cut-outs must be adjusted with sufficient precision to ensure that there is some friction when the tube #22 is inserted. Undersize them and adjust them with a file if necessary.
iv. Cut a 2.5 m length of int ø 72.5 mm/ext ø 76 mm PVC pipe (tube #22).
v. Drill several ø 5 mm holes between approximately 50 to 70 mm from the lower (Fig. 18*b*) end of tube #22.
vi. Drill four ø 4 mm holes at 90° from each other and ∼500 mm from the upper edge of tube #22 and screw in stop screws for the gauge unit;
vii. Insert tube #22 into the #23–#24-#25 assembly (Fig. 16*c*) down to the point where it stops on the heads of the screws added in step (vi) above;
viii. Float: cut a 120 mm length of int ø 55 mm/ext ø 60 mm PVC pipe (tube #19).
ix. Seal a PVC end cap (#18; 60/67 mm inner/outer ø) to one end of tube #19 with silicone sealant.
x. Weigh and pour ∼200 g of sand (or the mass necessary required to bring the top of the float to within 3 cm of the water surface (Fig. 17)) in the container thus formed (#18 + #19) and seal second PVC end cap (#20) to the other end of tube #19 with silicone sealant.

i. Cut a 1.8 m length of int ø 132 mm/ext ø 140 mm PVC pipe (tube #25).
ii. Drill regularly spaced ø 5 mm holes all around tube #25 circumference in the manner of a strainer tube (Fig. 16*a,b*).

**Fig. 17:**
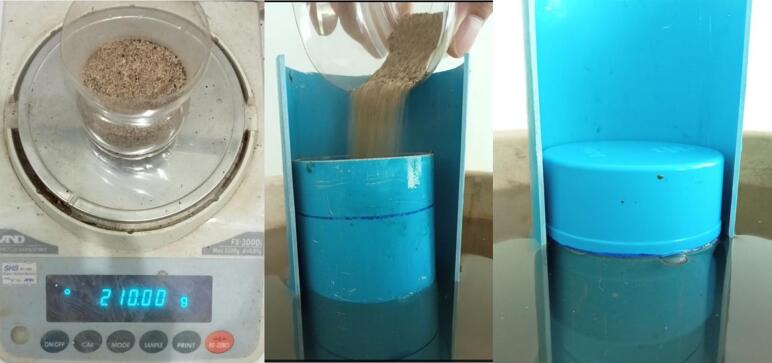
Preparing the float; a. Weigh the estimated necessary mass of sand; b. Pour sand into the container formed by the PVC tube #19 and sealed end cap #18 until the desired floating line is reached; c. Seal top end cap #20 to finalize the float.
xi. Prior to inserting the float in tube #22, check centering of the laser pointer; to this end, prepare a sheet of cardboard on which a circle of the same diameter as that of tube #22 is drawn; insert the gauge unit at the top end of #22 until it rests on the tips of stop screws added at step (vi) above (Fig. 18*a*); Initiate the BLE connection by pressing the RESET push-button and connect to the device using the Serial Bluetooth Terminal app; Turn on the laser beam by entering the lO command; match the circle drawn on the cardboard sheet with the lower end of tube #22 (Fig. 18*b*) and move it away gently and vertically in the downward direction to check that the beam is as close as possible to the middle of the circle (Fig. 18*c*). Turn OFF the beam using lC command.

**Fig. 18:**
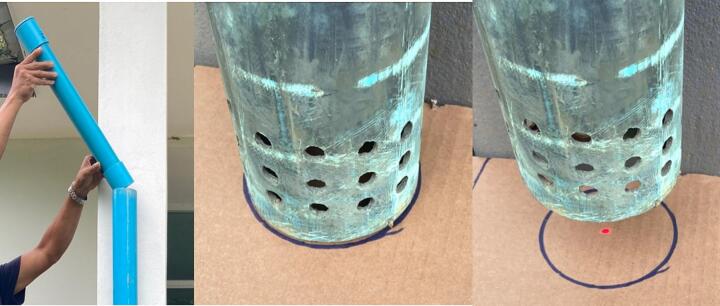
Checking alignment of the distance meter laser beam. a. Insert the gauge unit at the top end of float guide tube #22; b. Draw a circle of the same diameter as that of tube #22 on a sheet of cardboard; c. Gently move the cardboard sheet away from the end of the tube to check that the beam is as close as possible to the centre of the circle.
xii. Insert the float through the lower end of tube #22 and screw in the float stop screw.
xiii. Put on the top cover #21 to the top of tube #22.

### 4.7 Field setup

**Fig. 19:**
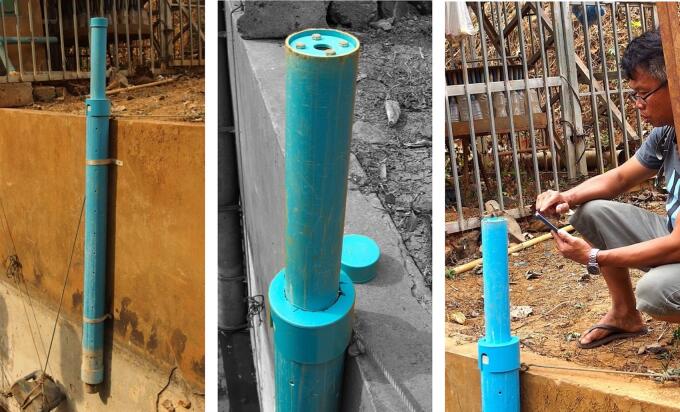
Field deployment of the water level gauge for water level monitoring in a weir; a. Overall view of the complete device installed on the wall of a weir using wall mounting brackets; b. Top view of the gauge unit inserted at the upper end of the float guide tube #22 which is itself inserted into the strainer tube #25, the removable end cap (#21) for #22 is taken out and placed on the edge of the wall allowing access to the push-button cover assembly; c. Data downloading in the field using the Bluetooth Low Energy connectivity with Android app ‘Serial Bluetooth Terminal’.

If the installation site includes a structure such as a wall, as in the case of our field trials in weirs, fasten the entire device (i.e. gauge unit inserted at the top of float guide tube + strainer tube) using at least two wall mounting pipe clamps of the correct diameter to firmly fix the strainer tube #25 in a vertical position (to be checked with a spirit level) (Fig. 19). If the device is to be installed in the bed of a watercourse or in any situation where no pre-existing support structure is available, drive a metal rail or tube at least 1 m deep into the ground, leaving a sufficient length above ground and securely fasten the strainer tube to the rail/tube using pole mount brackets or heavy-duty cable ties.

## 5 Operation instructions

### 5.1 Program description

**Fig. 20:**
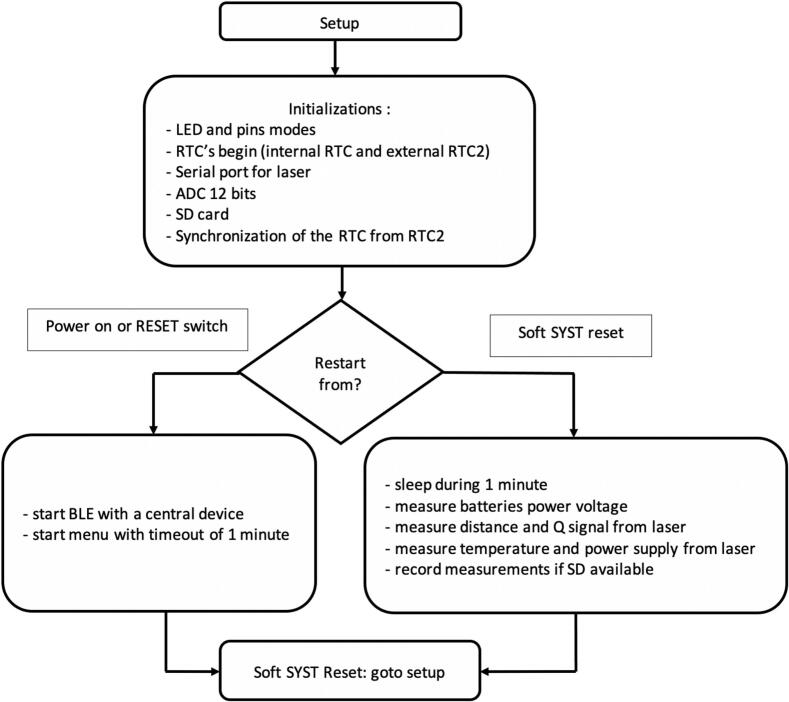
Flowchart of the water level gauge program.

The program used to operate the water level gauge was written with the open-source software IDE 1.8.16 from Arduino (https://docs.arduino.cc/); it supports data acquisition at a 1-min time step, data saving to SD memory, RTC management and MKR sleep mode of WIFI 1010 between measurements (Fig. 20). The iterative data acquisition sequence includes 1. Powering up the distance meter for distance and temperature measurements, 2. Saving the data on the SD memory card and 3. Putting the micro-controller to standby mode until the next iteration. Configuration is accessible via BLE communication (see section 6.3) using a smartphone running the ‘Serial Bluetooth Terminal’ Version 1.48 application for Android written by Kai Morich (available from Google Play Store or https://www.kai-morich.de/android/).

To load code onto the micro-controller card, connect the MKR WIFI 1010 to the computer with the USB / micro-USB and follow the instructions as described under section 5.4 *First startup.* Note that program uploading and first startup need to be completed prior to finalizing the assembly of the water level gauge while the MKR WIFI 1010 micro-USB socket remains accessible.

Required libraries are:

• FlashStorage.h (Ver 1.0.0)
• SdFat.h (Ver 2.2.2)
• RTCZero.h (Ver 1.6.0)
• Wire.h (Arduino IDE default library, 20/11/24 revision)
• RTClib.h (Ver 2.1.1)
• HardwareBLESerial.h (Ver 1.0.0)

Changes must be implemented to some of these libraries, namely:

• RTClib/scr/RTC_PCF8523.cpp and RTClib/scr/RTClib.h:
  a. Read battery backup status register
  b. Read Offset register
  c. Read/Write Minute alarm register
  d. Read/Write Control1 and Control2 registers
• HardwareBLESerial/scr/HardwareBLESerial.cpp and HardwareBLESerial/scr/HardwareBLESerial.h:
  e. Print of an unsigned char value
  f. Println of an unsigned char value

These changes have already been implemented in the versions of these files available in the project repository (https://data.mendeley.com/datasets/dsffp9psmy/1).

### 5.2 RTC clock drift and correction procedure

As the quartz crystal frequency of the RTC deviates from the nominal value of 32.768 kHz as temperature departs from 25 °C, the manufacturer recommends setting an offset (PCF8523 Real-Time Clock (RTC) and calendar).

Procedure:

i. Cancel the previous drift calibration (a command). Set precisely the D0 and T0 date and time (t command) using date and time from a reference clock (e.g. smart phone, computer connected to a NTP server).
ii. After a one week period, check the D1 and T1 date and time (d command) and compare them with the time from an internet time server. Calculate the drift ddd in seconds (+/-) (D + T) – (D1 + T1) and the observation period sssssss in seconds (D1 + T1) – (D0 + T0).
iii. Correct the RTC using the c command: csssssss + ddd with sssssss the observation period in seconds and the sign of the drift (+ if the RTC run faster than the reference, − if slower) and ddd the drift in seconds.
iv. Reset precisely the date and time with the t command according to procedure described above (a) and check the drift again after 1,2…7 days.

This procedure gives satisfactory results if the ambient temperature does not vary much from 25 °C. Outdoors, a more precise correction must be applied if the average daily temperature departs significantly from 25 °C [14]. For example, following successive periods of 72 h at 5 °C and 45 °C, we experimentally observed that the RTC module underwent a drift of 7 s (i.e. 20 ppm). Depending on the timestamp accuracy required, periodic offset corrections or time resetting might be needed.

### 5.3 BLE communication and data downloading

**Fig. 21:**
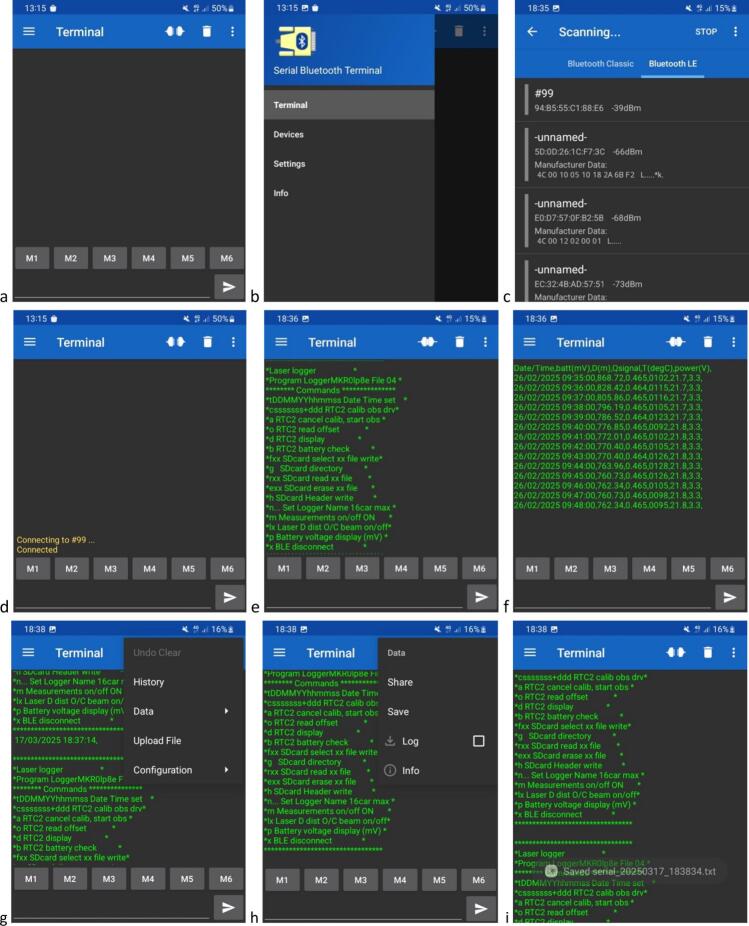
Screenshots of the’Serial Bluetooth Terminal’ application used for BLE communication with the of self-contained water level gauge; a. Main window; b, c. Device selection; d. Connection to device; e. Main menu of the water level gauge; f. Example of reading of data stored on the device’s micro-SD card; g,h,i. steps to follow to save data buffer.

The of self-contained water level gauge can be configured and its memory managed via Bluetooth Low Energy (BLE) communication to avoid the use of tools and cables in the field. On startup, the ‘*Serial Bluetooth Terminal*’ application displays a main window (Fig. 21*a*). To connect to the water level gauge, click on the icon at the top left, then select ‘*Devices*’ from the drop-down menu (Fig. 21*b*). Select the Bluetooth LE tab and press the device reset button. Tap on the name of the water level gauge which appears at the top of the list (with the highest RSSI value due to its proximity) (Fig. 21*c*). Successful connection is confirmed by an echo in the app’s main window and the change of the ‘*connect’* icon in the top ribbon (Fig. 21*d*). To access the water level gauge main menu, type any character that is not used as an input command, such as, for example’ 1′ or ‘w’. This triggers the display of the commands accepted by the device (Fig. 21*e*). If necessary, to improve the readability of the display, font size and type can be adjusted under the ‘*Settings’* menu. The list of available commands is given in Table 3. For example, to read data from the micro-SD card, type ‘rXX’ where XX is the name of the file to be read, which results in all the data stored in this specific file to be displayed on the app’s main window (Fig. 21*f*). Optionally, under the ‘*Send’* tab, it is possible to adjust ‘*Local echo’* and to ‘*Clear input on send’* as desired. Note that while communicating with the device via BLE, measurements are suspended. BLE connection is automatically stopped and measurements resumed if no commands are initiated via the Serial Bluetooth Terminal’ application within 1 min.

**Table 3:** Available commands (transmitted by clicking the arrow at the bottom right of the’Serial Bluetooth Terminal’ window).

tDDMMYYhhmmss	Date and Time set (total exactly 13 digits: if for example date is 3 January 2025 9:3:30, enter t030125090330).
csssssss + ddd	Drift correction of the external RTC2 (+- number of seconds (ddd 3 digits) / duration of observation (sssssss 7 digits, ideally 1 week)). Answer Y to confirm.
a	Cancel the drift calibration of the external RTC2 and restart observation.
o	Read the drift calibration offset of external RTC2.
d	Display of date and time: DD/MM/YYYY hh:mm:ss,
b	Check the coin cell battery voltage of the external RTC2 (ok or low).
fxx	Select file xx (00 to 99) to write
g	Listing of the files with Date/time of last access and size in bytes
rxx	Read the file xx.
exx	Erase the file xx. Answer Y to confirm.
h	Write a header on the SD.
n…	Set the name of the logger, 16 characters max.
m	Start / Stop the measurements at 1-minute step. ON/OFF is displayed accordingly.
lx	Do Laser command, x=: D distance/Q signal, O beam on (for positioning the beam on the target), C beam off, S temperature and power of the laser, M slow mode, F fast mode, V serial and software version numbers.
p	Battery power measurement in mV. Note that the value displayed is lower than that recorded during the laser measurement due to the BLE's power consumption.
x	Exit from the menu. Measurements resume automatically.

**Fig. 22:**
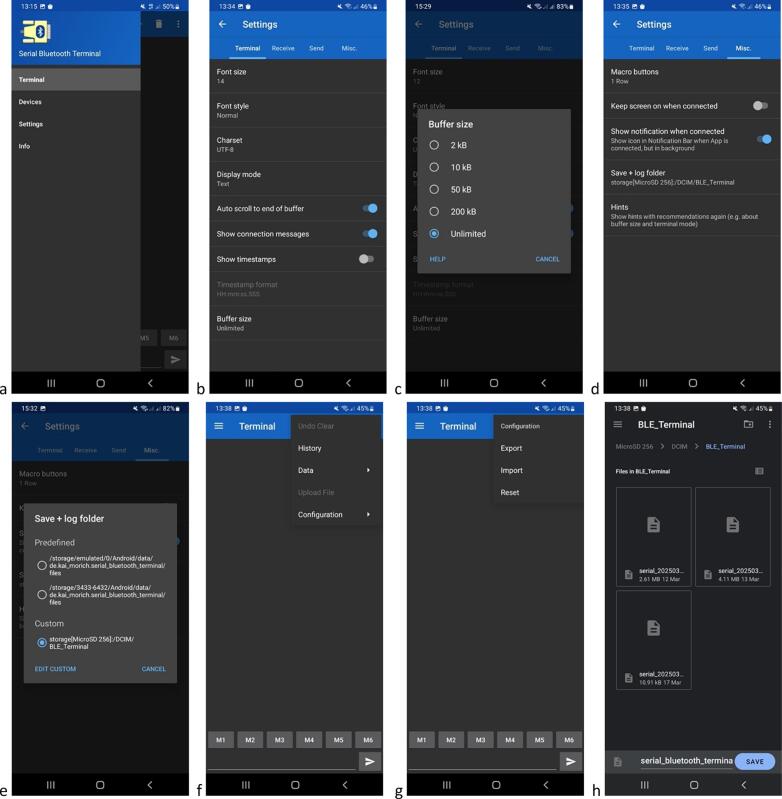
Screenshots showing the configuration of the’Serial Bluetooth Terminal’ application used for BLE communication with the self-contained water level gauge using an Android smartphone; a. Selecting ‘Settings’; b,c. Under the ‘Terminal’ tab, deselecting ‘Show timestamps’ and selecting ‘Buffer size: Unlimited’; d,e. Under the ‘Misc.’ tab, deselecting ‘Keep screen on when connected’ and selecting ‘Show notification when connected’, choosing ‘Save + log folder’ to set the local file path where the data buffer is to be saved; f, g, h. Access saved files by clicking on the top right three vertical dots of the top ribbon, then ‘Configuration > Export’.

To save data in a local file on the Android phone or tablet, the application must be configured using the drop-down menu at top left, by selecting ‘Settings*’* (Fig. 22*a*), then, under the ‘*Terminal’* tab, deselecting ‘*Show timestamps’* (Fig. 22*b*) and selecting ‘*Buffer size: Unlimited’* (Fig. 22*b,c*); under the ‘*Misc.’* tab, deselect ‘*Keep screen on when connected’* (Fig. 22d); select ‘*Show notification when connected’* and tap on ‘*Save +* log *folder’* (Fig. 22*d*) and choose a local file path where the data buffer will be saved (either on the phone’s internal memory or micro-SD card) (Fig. 22*d,e*). It is then possible to visualize the destination folder from the app main window by clicking on the top right three vertical dots of the top ribbon and then ‘*Configuration* > *Export’* (Fig. 22*f,g*). Data are saved to a *serial_yyyymmdd_hhmmss.txt* file in text (Fig. 22*h*), comma separated format.

Once the application has been configured, the data buffer downloaded in the field *via* BLE can be saved to a local file on the phone, from the terminal's main screen, by clicking on the three vertical dots at the right of the top ribbon, then ‘Data’ > ‘Save’ (Fig. 21*g,h*), with data saving confirmed by a character string ‘Saved *serial_yyyymmdd_hhmmss.txt’* that appears superimposed towards the bottom of the window (Fig. 21*i*).

When the gauge housing is taken back from the field for battery replacement the files can be read directly from the micro-SD card using a micro-SD card reader.

## 6 Validation and characterization

### 6.1 Distance meter sensitivity to temperature

**Fig. 23:**
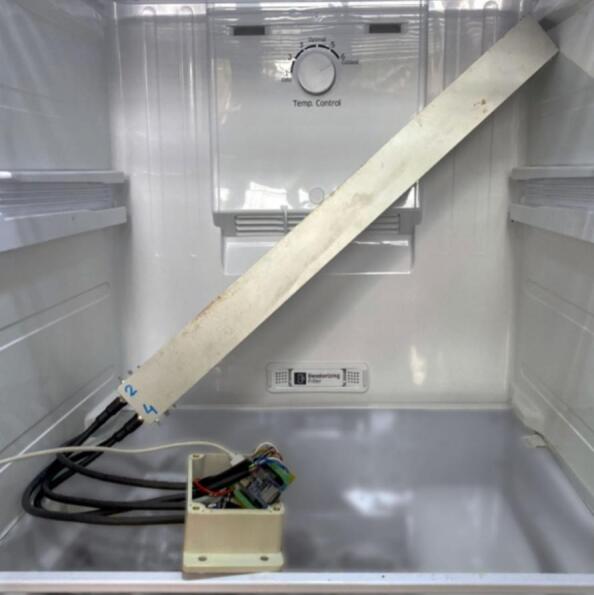
Picture showing the enclosure containing the four tested units while being submitted to 0–5 °C temperatures in a fridge.

**Fig. 24:**
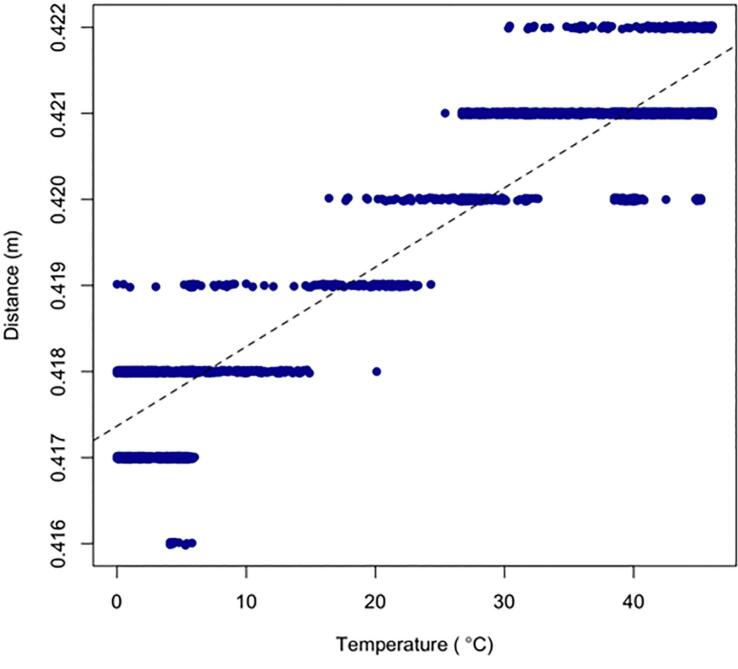
Distance measurements obtained with one JRT-M703A distance meter at 6 s time intervals over a period of approx. 7 h and for a temperature ranging of 0 to 45 °C.

To estimate the stability of the distance measurement values yielded by the JRT- M703A laser distance meter relative to ambient temperature variations, we carried out some laboratory tests using four JRT- M703A units, encapsulated at one end of a 50 cm long aluminum enclosure, and aiming at the other end of the enclosure, taking distance measurements with a time step of 6 s over a period of approx. 7 h, at temperatures ranging from 0 to 45 °C (Fig. 23*,* Fig. 24.).

While two of the four devices tested showed distance measurements that correlated well with ambient temperature, with a linear correlation coefficient r^2^ > 0.88, the other two devices behaved more erratically. Notwithstanding the differences in distances indicated by the four distance meters and likely linked to the construction of the enclosure, this test showed that the units were somewhat sensitive to ambient temperature, especially as the temperature range considered was wide. When the full 45 °C range was considered, 90 % of the measurements fell within ±2 mm of the median distance value whereas when only the 20 to 30 °C range was considered, 90 % of the measurements fell at most within ±1 mm of the median. This test allowed us to conclude that at ambient temperatures common in our field conditions, the JRT-M703A units can produce distance measurements with an accuracy of ±1 mm, which is within the manufacturer's specification (Table 4).

**Table 4:** Influence of temperature on distance readings, 5th, 50th and 95th percentile for 3 temperature ranges and four JRTM703A units.

		Percentile		
	Distance meter	5 %	Median	95 %
T [0–45 °C]	D1	421	424	425
	D2	420	421	423
	D3	416	418	420
	D4	417	421	422

T [15–35 °C]	D1	423	425	425
	D2	420	422	423
	D3	418	418	420
	D4	419	421	421

T [20–30 °C]	D1	424	425	426
	D2	422	423	423
	D3	418	418	419
	D4	419	420	421

### 6.2 Energy balance and autonomy

From direct measurements of the operating device, the typical consumption of the water level gauge over a period of 1 h (without taking into account BLE connections) was found to be: 2.377 + 0.428 + 0.006 = 2.811 mAh (Table 5). With a set of 12,000 mAh batteries, a theoretical maximum autonomy of 12,000/2.811 = 4,269 h = 177.9 days, i.e. of the order of 6 months, can be expected. Note that additional power drainage will result from turning on the BLE in the field to download data. For example, a 5-minute download will result in – 17.5*(5/60) = 1.46 mAh. Under field conditions, with night-time temperatures falling to around 10 °C we were able to verify a real-life autonomy of at least 2 months.

**Table 5:** Typical current consumption with data measurement and saving every minute, measured directly between the + of battery pack and Vin of the MKR board.

Mode	Standby	Measurement	Data saving	BLE
Current (mA)	2.41	32.1	25.5	17.5
Duration (s)	59.185	0.8	0.015	
Power drainage (mAh)	2.41*(60–0.8–0.015)/60 = 2.377	32.1*0.8/60 = 0.428	25.1*0.015/60 = 0.006	

### 6.3 Device qualification

The automatic water level gauge has been qualified against the SE200 water level meter from OTT (https://www.otthydromet.com/en/), a commercial device that uses a float linked to a shaft encoder, i.e. a rotating shaft connected to an electronic system that provides information about the rotation of the shaft in relation to the position of the float. Although the SE200 is widely regarded as an accurate, precise and reliable device for the continuous measurement of water level, it is based on a different technology to our self-contained water level gauge and has moving mechanical parts that are susceptible to rust and insect damage, which is not the case with our device. For this qualification exercise, comparisons between the SE200 and our self-contained water level gauge were conducted in the laboratory and in the field.

#### 6.3.1 Laboratory testing

**Fig. 25:**
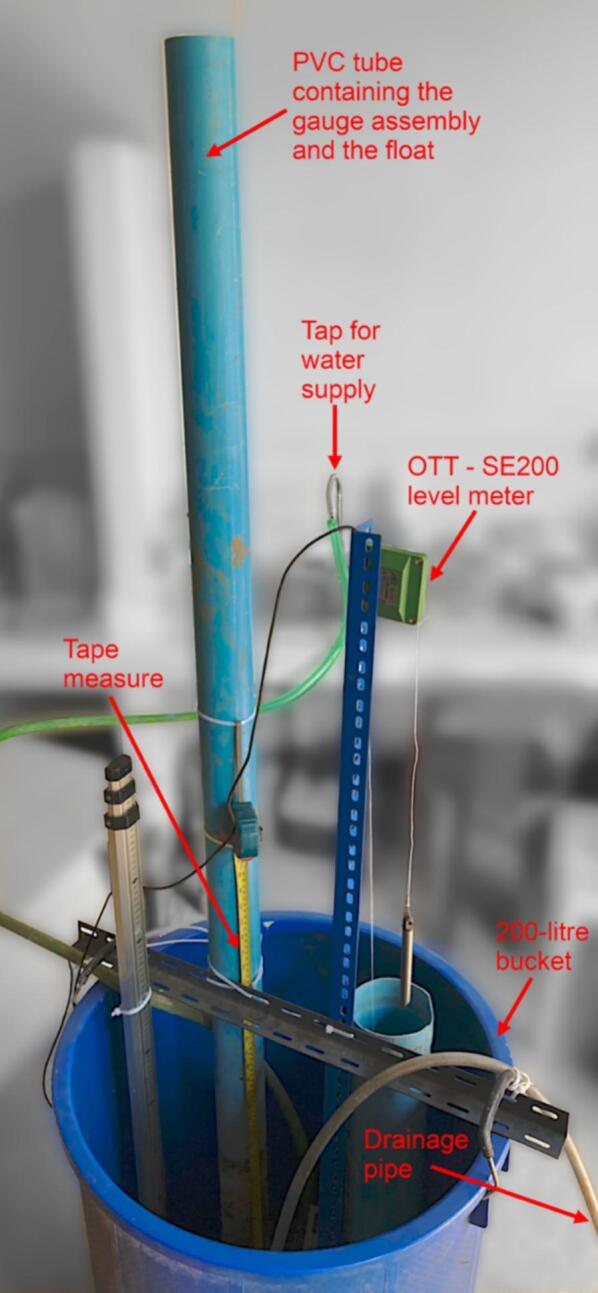
Picture of the experimental setup used to compare the water levels measured using the self-contained water level gauge with that measured manually using a ruler and with values from the OTT-SE200 water level meter.

**Fig. 26:**
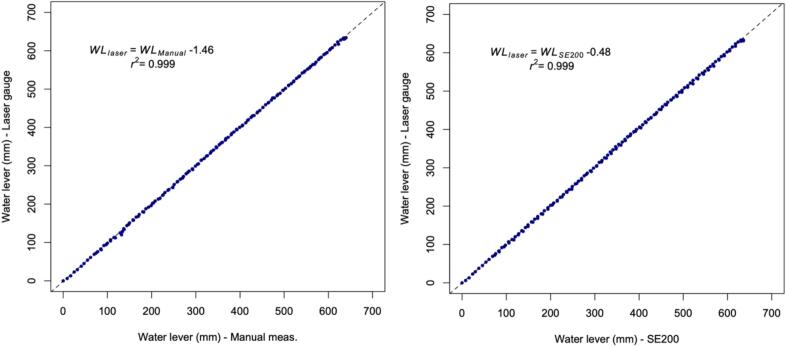
Comparison between the water level measured using the self-contained water level gauge and manual measurements (left panel) or OTT-SE200 water level meter (right panel) through an experimental filling-emptying cycle of an amplitude of 600 mm with rising and receding rates of ∼10 mm. min^−1^.

A metal rail fixed horizontally between the edges of a 200-litre bucket was used to support a PVC tube to which a metal ruler was attached and into which the self-contained water level gauge was inserted, as well as a vertical shelf rail to which the SE200 was attached and a PVC tube in which its float and counterweight connected by a tether could freely move (Fig. 25). The SE200 was connected to a Campbell CR200 data logger (https://www.campbellsci.com/) to measure and record the water level every minute (Fig. 26). At the onset of the experiment, date and time were synchronized between the CR200 and the self-contained water level gauge. Both devices were set to the same initial reference before filling the bucket and the cable wound on the SE200′s encoder wheel so that both devices recorded directly comparable water level variations. A filling-emptying cycle of an amplitude of 600 mm with rising and receding rates of ∼10 mm. min^−1^ mimicking as closely as possible a typical flood was carried out.

This laboratory test showed that the three measurement methods, namely, manual readings using a ruler, the SE200 water level meter and the self-contained water level gauge gave highly correlated results, with a slope and a linear correlation coefficient both very close to 1, with a 0.9 probability of measurements from the self-contained water level gauge to fall within 3 and 5.5 mm of manual and SE200 measurements, respectively (corresponding to residual standard errors of 2.16 and 2.71).

#### 6.3.2 Field testing

**Fig. 27:**
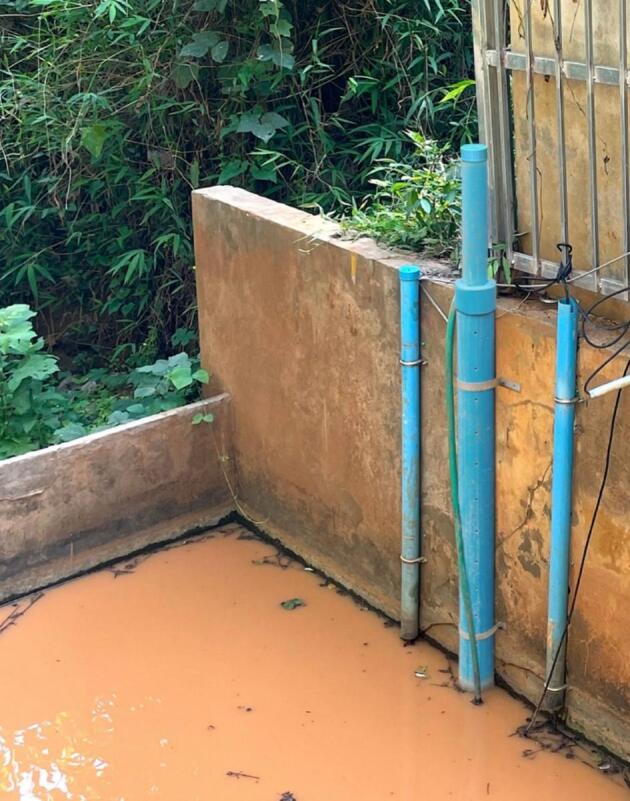
Field deployment of the self-contained water level gauge at the M−TROPICS critical zone observatory during the 2024 monsoon.

**Fig. 28:**
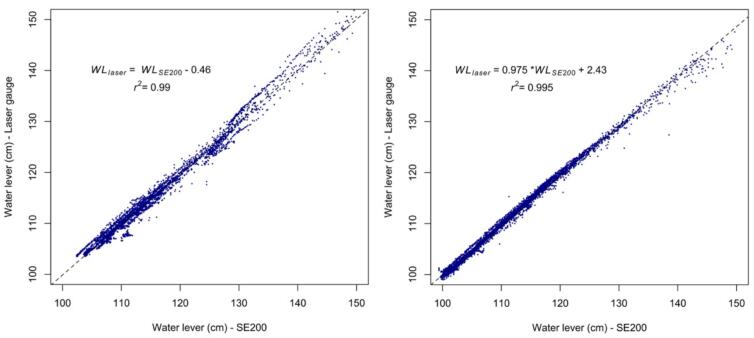
Field comparison of the performance of the self-contained water level gauge with that of the OTT-SE200 water level instrument during the 2024 rainy season in the M−TROPICS experimental catchment, northern Laos. Left panel results for upstream gauging station (19°51′37.3″N 102°10′21.6″E, elevation 538 m); right panel: results from downstream gauging station (19°51′29.6″N 102°10′12.5″E, elevation 521 m).

To assess the performance of the self-contained water level gauge under field conditions, we deployed it at two gauging stations part of a long-term environmental observatory in a tropical mountain area of northern Lao PDR [3]. At each station, a self-contained water level gauge and a SE200 sensor were installed alongside each other (Fig. 27). Data were collected from May to October 2024 and included a number of flood events typical of the rainy season in this area. Based on this field testing, the two devices appear to perform similarly, with a slope and linear correlation coefficient both very close to 1 (Fig. 28).

Under these field conditions the largest differences between measurements taken by the two devices were observed for the highest water level values and/or the fastest rates of change in water level. Overall, 87.5 % of the measurements taken by the two devices were within 10 mm of each other, corresponding to a residual mean standard error (RMSE) and a mean absolute error (MAE) of 9.9 and 2.6 mm, respectively (n = 8017), over a measurement range of 0.5 m.

#### 6.3.3 Further developments, improvements and applications

• While this paper only presents the application of the stand-alone gauge for water level monitoring in a weir, the design chosen is versatile enough so that it can be applied to other applications such as groundwater level monitoring in piezometers or rainfall monitoring in a totalizer rain gauge
• The autonomy of the stand-alone water level gauge presented in the paper is already substantial, further reduction of power requirements are possible through the implementation of Very low power (VLP) circuit (see file ‘SCWL gauge electronic design April 2025.pdf’ in the project repository): in this configuration the MKR WIFI 1010 and the MKR MEM are powered through a MOSFET transistor triggered using the SQW signal interrupt from the RTC. BLE connection is activated by pushing the (ON)-OFF push-button connected to SQW, not to RESET. During the standby mode, the current fall to 2.1 µA.
• Regarding the RTC drift correction discussed in this paper, the procedure could be automated with the addition of a GPS module that would retrieve a reference time at regular intervals and adjust the RTC module time accordingly.
• Finally, although the stand-alone gauge has been designed with simplicity and autonomy in mind, some applications could certainly benefit from the addition of minimal LORA / GSM telemetry as a means to remotely assess the status of the device (for example, the number of measurements taken and battery voltage could be forwarded remotely on a daily basis).

## CRediT authorship contribution statement

**Alain Pierret:** Writing – review & editing, Writing – original draft, Visualization, Validation, Methodology, Funding acquisition, Formal analysis, Conceptualization. **Norbert Silvera:** Writing – review & editing, Writing – original draft, Visualization, Software, Methodology, Conceptualization. **Keo Oudone Latsachack:** Resources, Investigation, Data curation, Conceptualization. **Khampasith Chanthavong:** Software, Resources. **Phabvilay Sounyafong:** Resources, Investigation, Data curation. **Olivier Ribolzi:** Writing – review & editing, Writing – original draft, Visualization, Validation, Supervision, Project administration, Methodology, Funding acquisition, Formal analysis, Conceptualization.

## Declaration of competing interest

The authors declare that they have no known competing financial interests or personal relationships that could have appeared to influence the work reported in this paper.
